# HIV Transmission in a State Prison System, 1988–2005

**DOI:** 10.1371/journal.pone.0005416

**Published:** 2009-05-01

**Authors:** Krishna Jafa, Peter McElroy, Lisa Fitzpatrick, Craig B. Borkowf, Robin MacGowan, Andrew Margolis, Ken Robbins, Ae Saekhou Youngpairoj, Dale Stratford, Alan Greenberg, Jennifer Taussig, R. Luke Shouse, Madeleine LaMarre, Eleanor McLellan-Lemal, Walid Heneine, Patrick S. Sullivan

**Affiliations:** 1 Division of HIV/AIDS Prevention, National Center for HIV/AIDS, Viral Hepatitis, STD and TB Prevention, Centers for Disease Control and Prevention, Atlanta, Georgia, United States of America; 2 Epidemiology Program Office, Office of Workforce and Career Development, Centers for Disease Control and Prevention, Atlanta, Georgia, United States of America; 3 Department of Epidemiology and Biostatistics, George Washington University School of Public Health and Health Services, Washington, D. C., United States of America; 4 Georgia Department of Human Resources, Division of Public Health, Atlanta, Georgia, United States of America; 5 Georgia Department of Corrections, Atlanta, Georgia, United States of America; McGill University Health Center, Montreal Chest Institute, Canada

## Abstract

**Introduction:**

HIV prevalence among state prison inmates in the United States is more than five times higher than among nonincarcerated persons, but HIV transmission within U.S. prisons is sparsely documented. We investigated 88 HIV seroconversions reported from 1988–2005 among male Georgia prison inmates.

**Methods:**

We analyzed medical and administrative data to describe seroconverters' HIV testing histories and performed a case-crossover analysis of their risks before and after HIV diagnosis. We sequenced the *gag*, *env*, and *pol* genes of seroconverters' HIV strains to identify genetically-related HIV transmission clusters and antiretroviral resistance. We combined risk, genetic, and administrative data to describe prison HIV transmission networks.

**Results:**

Forty-one (47%) seroconverters were diagnosed with HIV from July 2003–June 2005 when voluntary annual testing was offered. Seroconverters were less likely to report sex (OR [odds ratio] = 0.02, 95% CI [confidence interval]: 0–0.10) and tattooing (OR = 0.03, 95% CI: <0.01–0.20) in prison after their HIV diagnosis than before. Of 67 seroconverters' specimens tested, 33 (49%) fell into one of 10 genetically-related clusters; of these, 25 (76%) reported sex in prison before their HIV diagnosis. The HIV strains of 8 (61%) of 13 antiretroviral-naïve and 21 (40%) of 52 antiretroviral-treated seroconverters were antiretroviral-resistant.

**Discussion:**

Half of all HIV seroconversions were identified when routine voluntary testing was offered, and seroconverters reduced their risks following their diagnosis. Most genetically-related seroconverters reported sex in prison, suggesting HIV transmission through sexual networks. Resistance testing before initiating antiretroviral therapy is important for newly-diagnosed inmates.

## Introduction

The estimated prevalence of HIV infection in the United States is more than five times higher among state prison inmates (1.9%) than for the general population (0.37%) [1], [2]. Although most inmates with HIV are infected before they enter prison, HIV risk behaviors, and occasionally HIV infection, during incarceration have been reported [3]–[7]. However, sparse information is available on HIV transmission within large state prison systems in general and on inmates' risk modification after HIV diagnosis, HIV transmission networks, or antiretroviral drug resistance in particular.

During 2004–2006, the Centers for Disease Control and Prevention (CDC), the Georgia Division of Public Health (GDPH), and the Georgia Department of Corrections (GDC) conducted an epidemiologic investigation of all 88 known HIV seroconversions identified among male GDC prison inmates since mandatory HIV screening for all new inmates was initiated in 1988. No seroconversions were reported among female inmates. In a previous case-control study among GDC male inmates, we found that sex in prison, tattooing in prison, black race, and a body mass index of 25.4 kg/m2 or less were significantly associated with HIV seroconversion [7].

In this paper, we follow up on our previous report by combining data from medical and administrative records, behavioral risk interviews, and genetic analysis of seroconverters' HIV strains to describe HIV transmission networks within the GDC prison system. We use interview data to describe self-reported risk modification among seroconverters after their HIV diagnosis. Because tattooing—which was associated with HIV seroconversion in the initial case-control study—is an unproven mode of HIV transmission, we re-interviewed seroconverters who initially reported tattooing as their only potential risk for HIV infection.

## Methods

### GDC intake and HIV testing processes

In Georgia, inmates charged with a felony offense are housed in local or county jails while awaiting sentencing. Most jails do not offer HIV testing to inmates. After sentencing, inmates are transferred to a GDC reception center for security classification and an entry medical evaluation and then to one of 73 GDC facilities. In July 1988, GDC initiated mandatory HIV screening of all inmates during their entry medical evaluation. Voluntary annual HIV testing was offered to inmates from July 2003–June 2005. Inmates are also tested upon their request, if clinically indicated, upon a court order, or following an incident involving their exposure to blood or body fluids; they are not tested before release.

### Definitions

Duration of incarceration was defined as the time an inmate remained in continuous correctional custody from his jail entry date through his release from prison, death, or the last date of data collection, whichever occurred earlier; seroconversion as at least 1 negative HIV test result after the start of incarceration followed by a confirmed positive HIV test result during the same incarceration; movement as an inmate's transfer between correctional facilities (jails, county prisons, state prisons, private prisons, transitional centers); and a facility where HIV infection definitely occurred as one in which a seroconverter had a negative HIV test result followed by a confirmed positive result during the same stay at that facility.

### Procedures

#### Recruitment

We recruited male seroconverters aged 18 years or older from 31 GDC facilities where seroconverters resided from February 2005–March 2005. All participants provided written informed consent for interviews and blood specimen collection. CDC determined that these activities, as a public health response to a request to investigate HIV transmission in a state prison system, did not require institutional review board approval under human-subjects protection guidelines, and the state's Institutional Review Board of record concurred.

#### Seroconverters' Characteristics

We reviewed GDC medical and administrative data to describe seroconverters' demographic characteristics, number of previous prison incarcerations, offense type, and year and duration of current incarceration.

#### HIV Infection During Incarceration

To confirm that the reported seroconversions occurred during incarceration, we reviewed GDC medical and administrative data to calculate, for each seroconverter, the number of negative HIV test results and the time from the start of incarceration to the first negative result and to the positive result. We also examined seroconverters' movement histories to identify the facility where the infection occurred.

#### HIV Risk Behaviors

We used Questionnaire Development System v2.1 software (Nova Research, Bethesda, MD) to develop and administer audio computer-assisted self-interviews (ACASI) to collect data on seroconverters' risk behaviors before and during incarceration. We asked 74 seroconverters incarcerated at the time of the investigation to participate in these interviews and obtained written informed consent from all of them. Two seroconverters had died and we did not attempt to contact the 12 seroconverters who had been released. To determine if knowledge of HIV infection was associated with a reduction in risk behaviors, we performed a matched-pairs case-crossover analysis of inmates' self-reported risk behaviors before and after HIV diagnosis. We used SAS v9.1 software (SAS Institute, Cary, NC) to calculate exact odds ratios (ORs) and 95% confidence intervals (CIs) and Fisher's exact test to determine whether differences in risks before and after HIV diagnosis were significant (*p* values<0.05).

For 12 seroconverters who reported in ACASI that tattooing was their only potential risk for HIV infection during incarceration, we conducted follow-up face-to-face structured interviews to elicit previously unreported risks. We asked for the names of sex, IDU, or tattooing contacts and offered HIV testing to these contacts. We did not conduct contact-tracing activities for seroconverters who reported an established risk for HIV transmission.

#### HIV Transmission Networks

To identify genetically-related HIV strains, we collected plasma specimens from seroconverters who participated in ACASI and from contacts named by 2 or more seroconverters in follow-up interviews. We amplified the p17 *gag* gene (396 nucleotides) and a partial fragment of the gp41 *env* gene (360 nucleotides) of the HIV genome with nested polymerase chain reaction (PCR) procedures and sequenced them. We analyzed sequence data and constructed a neighbor-joining phylogenetic tree, using the general time reversible model of evolution, with the Phylogenetic Analysis Using Parsimony (PAUP) v.4.b10 program (Sinauer Associates, Inc., Sunderland, MA). The B.82CAN sequence (early, 1982 subtype B Canadian HIV strain) served as an outgroup sequence. We assessed support for internal nodes with the PAUP bootstrapping procedure and depicted internal nodes with bootstrap support of more than 85% in a phylogenetic tree.

We assigned seroconverters with genetically-related HIV strains to clusters and analyzed the combined cluster, inter-facility movement, and risk data of seroconverters and their named contacts to describe HIV transmission networks by the facility where transmission occurred and the possible mode of transmission. For the largest cluster, we created network diagrams with InFlow v3.0 software (Orgnet.com, Cleveland, OH) and connected each seroconverter in this cluster to all jails and GDC prisons in which he resided during his current incarceration. For each facility where a seroconverter in this cluster resided, we determined his HIV infection status while in that facility as either HIV-negative, unknown HIV status, new HIV diagnosis, or established HIV diagnosis. We eliminated all facilities linked to only one seroconverter to create a network of core GDC facilities, and assigned likely modes of HIV transmission on the basis of seroconverters' self-reported risk behaviors.

#### Drug-resistant Virus Transmission

To identify antiretroviral drug-resistance mutations and their transmission patterns, we used standard genotyping procedures on specimens collected from 67 seroconverters to sequence the protease and reverse transcriptase genes of the *pol* region (approximately 1,930 nucleotides) of their HIV strains. Additionally, we performed sensitive PCR-based assays for four major drug-resistant mutations (L90M in protease, and K70R, K103N, M184V in reverse transcriptase) on specimens collected from 13 antiretroviral-naïve (untreated) seroconverters and from two antiretroviral-treated seroconverters whose HIV strains did not show antiretroviral-resistance with standard sequencing methods [8]. We compared drug-resistant virus transmission among antiretroviral-treated seroconverters versus antiretroviral-naïve seroconverters, and among seroconverters with genetically-related HIV strains versus those with unrelated strains.

## Results

### Seroconverters' Characteristics

The median age of seroconverters was 24 years (range: 15–57 years) at the start of incarceration and 32 years (range: 21–58 years) at the time of HIV diagnosis; 59 (67%) were black and 29 (33%) white; 39 (44%) had been previously incarcerated in the Georgia state prison system a median of two times (range: 1–6 previous incarcerations); 76 (86%) had committed a violent offense, of whom 26 (34%) had committed a sexual offense (Table 1). Forty-five (51%) seroconverters entered prison during 1992–1998, and 30 (34%) were serving life sentences (Table 1). Seroconverters had spent a median of 124 days (range: 4–2437 days) in jail and a median of 7 years (range: 1–20 years) in prison before being diagnosed with HIV.

**Table 1:** Incarceration-related Characteristics of 88 Male Inmates (Seroconverters*) in Whom HIV Infection Was Diagnosed During Their Incarceration, Georgia State Prison System, United States, 1988–2005.

Characteristics	Seroconverters	
	n = 88	%
**Facility of infection** †		
Definite facility identified	36	41
Probable facility identified	24	27
Unknown facility of infection	28	32
**Incarceration**		
Previously incarcerated in Georgia prison system	39	44
Median # of previous incarcerations (range)	2 (1–6)	
Current incarceration: primary offense		
Violent offense‡	76	86
Sexual offense	26	30
Nonviolent offense§	12	14
Drug-related offense	2	2
Current incarceration start year		
1978–1984	6	7
1985–1991	22	25
1992–1998	45	51
1999–2003	14	16
Median duration of current sentence (range)∥	17 (2–57)	
≤5 years	8	9
6–10 years	16	18
11–20 years	27	31
>20 years	37	42
Life sentence	30	34

* A seroconverter is an inmate with ≥1 negative HIV test result followed by a confirmed positive HIV test result during his current incarceration.

† A definite facility of infection is one in which a seroconverter had a negative HIV test result followed by a confirmed positive result during the same stay at that facility; a probable facility of infection is one of two possible facilities where a seroconverter may have become infected based on his HIV testing history.

‡ Violent offenses include aggravated assault, armed robbery, attempted armed robbery, kidnapping, murder, robbery, vehicular homicide, voluntary manslaughter, and the sexual offenses of aggravated child molestation, aggravated sodomy, child molestation, rape, and statutory rape.

§ Nonviolent offenses include attempted burglary, burglary, conspiracy, motor vehicle theft, and selling or dealing of narcotics.

∥ Prison release dates for inmates eligible for parole correspond to their tentative parole month and year; actual prison release dates are used for former inmates who had been released, had died, or were on parole as of January 31, 2007; inmates serving a life sentence were assigned a sentence duration of 50 years.

### HIV Infection During Incarceration

**Figure 1:**
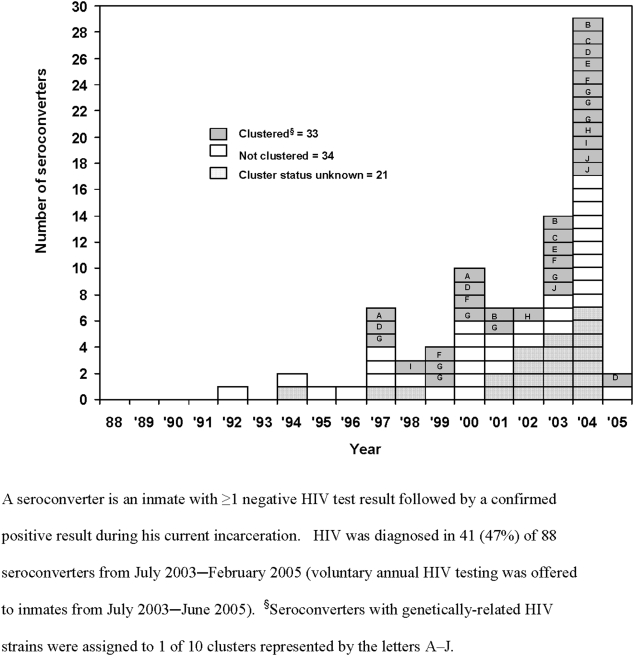
Year of HIV Diagnosis for 88 Male Inmates (Seroconverters) Who Became Infected with HIV During Incarceration, Georgia State Prison System, United States, 1988–2005.

All 88 known seroconverters were diagnosed with HIV from September 1992–February 2005; 41 (47%) from July 2003–February 2005, when GDC offered voluntary annual HIV testing (Figure 1). Seroconverters had a median of one negative HIV test result during incarceration (range: 1–7 negative tests); their first negative result was a median of 214 days (range: 7–4576 days) after the start of incarceration. The median duration from start of incarceration to HIV diagnosis was 8 years (range: 1–21 years). Of 47 seroconverters with a single negative HIV test result during incarceration, 26 (55%) were tested more than180 days after the start of incarceration, 15 (32%) 42–180 days after, and 6 (13%) less than 42 days after; 3 of these 6 reported having sex in prison before their HIV diagnosis and a fourth reported sex and IDU. We identified the GDC facility where HIV infection occurred for 36 (41%) seroconverters (Table 1).

### HIV Risk Behaviors

Of 74 seroconverters approached about participating in ACASI, 69 (93%) agreed to participate; of these, 49 (71%) reported sex, 4 (6%) reported IDU, and 36 (41%) reported tattooing in prison before their HIV diagnosis. In the case-crossover analysis (n = 66; three records had missing data and were excluded), we found that seroconverters were less likely to report sex (7 [11%] versus 49 [74%]) or tattooing (6 [9%] versus 36 [55%]) after their HIV diagnosis than before.

We re-interviewed 10 of the 12 seroconverters who originally reported tattooing as their only potential risk for HIV infection (1 refused; 1 was on parole and unreachable); 5 reported having sex with other male inmates during their incarceration and 5 denied having any other potential HIV risk. The 10 re-interviewed seroconverters named a total of 18 sex, IDU, or tattooing inmate contacts of whom 5 were known to be HIV-infected (including two seroconverters) and 2 could not be located; of the 11 remaining contacts who were offered HIV testing, 9 tested HIV-negative and 2 refused.

### HIV Transmission Networks

**Figure 2:**
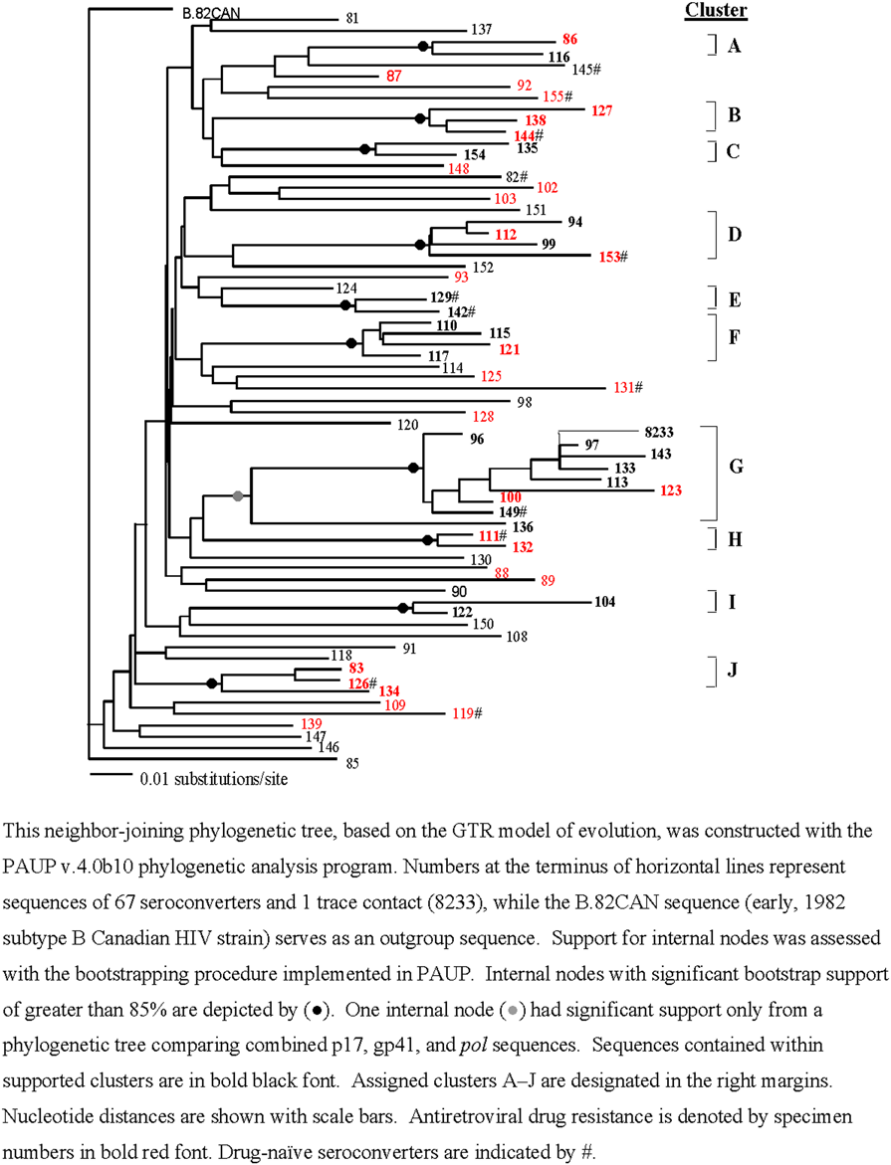
Genetic Analysis of Combined p17 and gp41 Sequences of HIV Strains from 67 Seroconverters and One Trace Contact, Georgia State Prison system, United States, 1988–2005.

Of 74 incarcerated seroconverters who were approached for blood specimens, 69 (93%) agreed to provide them; of these, two had unsuccessful blood draws. Genetic analysis of combined p17 *gag* and partial gp41 *env* sequences of 67 seroconverters' specimens demonstrated that 33 (49%) were associated in 10 distinct bootstrap-supported clusters (A–J) of 2–9 seroconverters each (Figure 2). These relationships were confirmed in further genetic analysis of *pol* sequences (n = 65, two specimens were refractory to amplification). Of the clustered seroconverters, 22 (67%) (including all seroconverters in clusters A, B, E, F, G, I, and J) had overlapping stays in the same prison with at least 1 other member of their respective cluster (Figure 2); 26 (79%) reported having sex (of whom 2 also reported IDU), 4 (12%) reported tattooing only, and 3 (9%) reported no risk behaviors.

**Figure 3:**
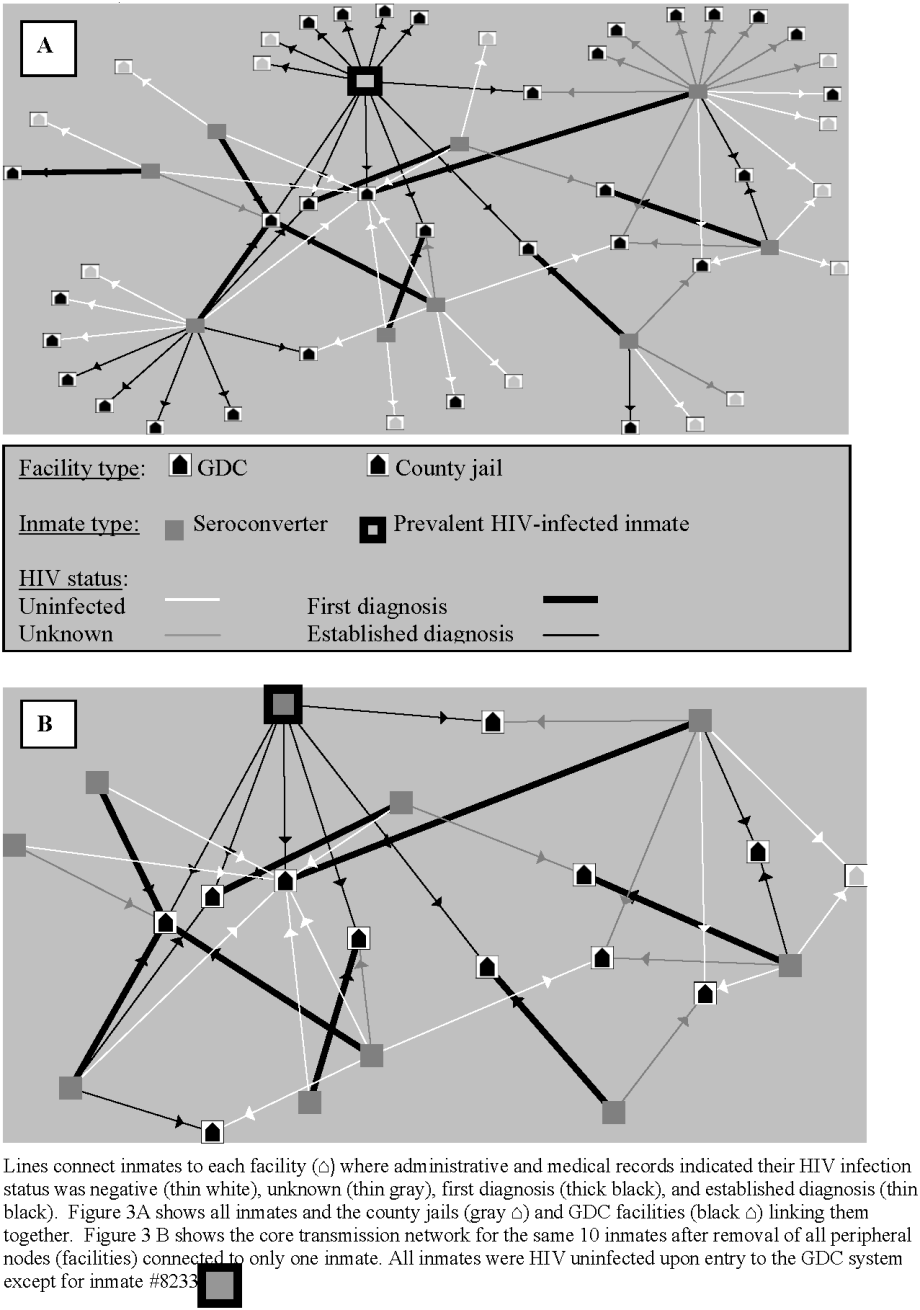
Transmission Network Diagrams Representing 10 Inmates (▪) from the Largest Genetic Cluster Detected During an Investigation of HIV Transmission Among 88 Inmates, Georgia State Prison system, United States, 1988–2005.

In cluster G, the single largest cluster (n = 9), all 4 seroconverters who reported having sex in prison before their HIV diagnosis named the same inmate as their sex contact; this inmate was infected with HIV before entering prison in 1994 and is therefore considered a possible index case. These four seroconverters, and four more in cluster G who reported tattooing as their only potential risk for HIV during incarceration, had overlapping prison stays with the possible index case; he reported tattooing as his only risk behavior while in prison ( Figure 2). A network diagram represents facility links for all nine members of cluster G and the possible index case (Figure 3).

### Drug-resistant Virus Transmission

Of 67 seroconverters whose blood samples were analyzed for antiretroviral drug resistance, 54 (81%) had received antiretroviral drug treatment and 13 (20%) were antiretroviral-naive. Twenty-nine (43%) seroconverters, including 21 who were treated and 8 who were antiretroviral-naïve, were infected with HIV strains resistant to one or more of the three classes of antiretrovirals including nucleotide reverse transcriptase inhibitors (NRTIs), non-nucleoside reverse transcriptase inhibitors (NNRTIs), and protease inhibitors (PIs). Of 21 treated seroconverters with antiretroviral-resistant HIV strains, 16 had single-class resistance (NRTI = 10, NNRTI = 6), 4 had dual-class resistance (NRTI and NNRTI), and 1 had triple-class resistance (NRTI, NNRTI, and PI). Of 13 antiretroviral-naïve seroconverters, 8 (62%) had single-class resistance (RTI = 4, NNRTI = 3, PI = 1). We found similar mutations in samples from both antiretroviral-naïve and treated seroconverters in clusters B, H, and J (Figure 2).

## Discussion

We found that 80% of seroconverters who were interviewed reported sex or IDU in prison before their HIV diagnosis and we identified 10 genetically-related clusters; two-thirds of seroconverters in these clusters had overlapping stays in the same prison with another member of their cluster. A high proportion of both antiretroviral-treated and antiretroviral-naïve seroconverters were infected with drug-resistant HIV strains.

Our findings highlight the importance of timely diagnosis and appropriate treatment of HIV-infected inmates. The time between HIV infection and diagnosis may have been shortened if routine voluntary testing was available for more than two of the 18 years since GDC first started testing inmates for HIV (half of all known seroconverters were diagnosed during the 2-year period when voluntary annual testing was offered to inmates). Frequent testing would also have made it easier to more accurately estimate the approximate period between infection and HIV diagnosis, and thereby would have minimized misclassification of in-prison versus pre-incarceration HIV acquisition.

The significant reduction in sex and tattooing among infected inmates following their HIV diagnosis is consistent with reports of reduced risk behaviors among nonincarcerated people after learning they are HIV-infected [9]. Most contacts named in follow-up interviews were reached and tested for HIV, demonstrating the utility of partner notification and contact-tracing activities in correctional settings.

Although HIV testing and risk data for seroconverters suggest that most were infected while incarcerated in GDC facilities, some may have been infected before incarceration or during initial jail stays before their transfer to a GDC prison. In particular, six seroconverters who reported unprotected sex with multiple female partners in the 6 months before incarceration and who had a single negative HIV test result within 42 days of being incarcerated may have had insufficient HIV antibodies to test positive at that time. However, three of the six reported having sex during incarceration before their HIV diagnosis. Furthermore, a quarter of all seroconverters traveled from prison to jail and back during their incarceration for varying lengths of time, and some of them may have become infected during these jail stays. Recall bias remains an important limitation in eliciting reports of risk behaviors in this population.

We previously reported that nine percent of all known HIV-infected male GDC inmates became infected in GDC facilities [7]. However, the actual percentage is likely to be higher because GDC offered voluntary annual HIV testing for only 2 years and does not test inmates prior to release. A comprehensive strategy to test inmates for HIV on entry, periodically during incarceration, and before release, as is recommended by CDC, would enable correctional systems to identify newly infected inmates and provide them with treatment and prevention services in a timely manner, as well as provide more accurate data for HIV incidence estimates.

The observed genetic clustering, the limited number of viral strains in circulation, and reports of sex among seroconverters with genetically-related HIV strains suggests that HIV was transmitted largely through sexual networks. By correlating administrative, risk, HIV testing, and contact-tracing data, we identified a sexual network for the largest genetic cluster; overlapping prison stays among seroconverters in other clusters suggest that other sexual networks existed. IDU did not appear to play a major role in HIV transmission, although inmates may have under-reported this risk behavior. Because only half of the seroconverters who initially reported tattooing as their only possible risk for HIV acquisition subsequently reported having had sex, we were unable to categorically rule out tattooing as a potential route of HIV transmission. However, while HIV can theoretically be transmitted via tattooing with non-sterile tattooing equipment, there is no documented case of HIV transmission via tattooing to date. The large proportion of inmates who reported sex and tattooing in prison indicates ACASI is a suitable method for eliciting sensitive information in correctional settings; however, all risk behaviors of interest are prohibited in GDC prisons and are thus likely to have been under-reported.

Drug-resistant HIV strains were found among both antiretroviral-naïve and antiretroviral-treated seroconverters. The 61% prevalence of such strains among antiretroviral-naïve seroconverters was substantially higher than that reported in other antiretroviral-naïve populations [10]. Moreover, five treated seroconverters were infected with HIV strains resistant to two or more classes of antiretrovirals. These findings suggest that complying with existing recommendations for antiretroviral drug-resistance testing before initiating a treatment regimen and then testing inmates periodically during their antiretroviral treatment are especially important in a prison setting. Ongoing counseling to encourage inmates to initiate and continue antiretroviral therapy when indicated may optimize treatment response, reduce antiretroviral resistance, and reduce HIV transmission [11].

HIV prevention education for incoming inmates and reinforcement of HIV prevention messages for all inmates are essential to reducing HIV risk among prisoners. We reiterate the importance of evaluating HIV prevention interventions, such as condom distribution, in correctional settings [7], [12]. Moreover, HIV prevention counseling for HIV-infected inmates and a system that ensures their uninterrupted medical care upon release should be public health priorities [7], [13]. Finally, correctional agencies should partner with public health agencies to assess and improve existing HIV prevention and treatment programs for all inmates.
